# NRARP displays either pro- or anti-tumoral roles in T-cell acute lymphoblastic leukemia depending on Notch and Wnt signaling

**DOI:** 10.1038/s41388-019-1042-9

**Published:** 2019-10-04

**Authors:** Inês Pinto, Mafalda Duque, Joana Gonçalves, Padma Akkapeddi, Mariana L. Oliveira, Rita Cabrita, J. Andrés Yunes, Scott K. Durum, João T. Barata, Rita Fragoso

**Affiliations:** 1 Instituto de Medicina Molecular João Lobo Antunes, Faculdade de Medicina Universidade de Lisboa, Lisboa, Portugal; 2 Centro Infantil Boldrini, Campinas, SP Brazil; 3 Department of Medical Genetics, Faculty of Medical Sciences, University of Campinas, Campinas, SP Brazil; 4 Cancer and Inflammation Program, Center for Cancer Research, National Cancer Institute, National Institutes of Health, Frederick, MD USA

**Keywords:** Acute lymphocytic leukaemia, Cell signalling, Cancer genetics, Oncogenes

## Abstract

T-cell acute lymphoblastic leukemia (T-ALL) is an aggressive hematological malignancy with a dismal prognosis in patients with resistant or relapsed disease. Although NOTCH is a known driver in T-ALL, its clinical inhibition has significant limitations. Our previous studies suggested that NRARP, a negative regulator of Notch signaling, could have a suppressive role in T-ALL. Here, we report that NRARP levels are significantly increased in primary T-ALL cells suggesting that NRARP is not sufficient to block NOTCH oncogenic signals. Interestingly, although NRARP overexpression blocks NOTCH1 signaling and delays the proliferation of T-ALL cells that display high levels of Notch1 signaling, it promotes the expansion of T-ALL cells with lower levels of Notch1 activity. We found that NRARP interacts with lymphoid enhancer-binding factor 1 (LEF1) and potentiates Wnt signaling in T-ALL cells with low levels of Notch. Together these results indicate that NRARP plays a dual role in T-ALL pathogenesis, regulating both Notch and Wnt pathways, with opposite functional effects depending on Notch activity. Consistent with this hypothesis, mice transplanted with T-cells co-expressing NOTCH1 and NRARP develop leukemia later than mice transplanted with T-NOTCH1 cells. Importantly, mice transplanted with T-cells overexpressing NRARP alone developed leukemia with similar kinetics to those transplanted with T-NOTCH1 cells. Our findings uncover a role for NRARP in T-ALL pathogenesis and indicate that Notch inhibition may be detrimental for patients with low levels of Notch signaling, which would likely benefit from the use of Wnt signaling inhibitors. Importantly, our findings may extend to other cancers where Notch and Wnt play a role.

## Introduction

T-cell acute lymphoblastic leukemia (T-ALL) is an aggressive hematological malignancy that accounts for 15 and 25% of pediatric and adult acute lymphoblastic leukemia, respectively [1]. Although the outcome of T-ALL patients has improved significantly, the prognosis of patients with resistant or relapsed disease remains dismal [2, 3]. This and the high toxicity of the existing therapies underline the need to develop more specific and effective therapeutic strategies [3, 4].

Notch signaling plays a fundamental role in the pathogenesis of T-ALL, with more than 50% of human T-ALL patients displaying NOTCH activating mutations [5]. The prominent role of Notch in the pathogenesis of T-ALL led to the clinical inhibition of this pathway [6, 7]. However, the strategies developed so far lack sufficient antileukemia effects and have been associated with high toxicity [6, 7]. Thus, a better understanding of the oncogenic mechanisms associated to Notch function and regulation in T-ALL is necessary.

NOTCH regulated ankyrin repeat protein (NRARP) is a transcriptional target of NOTCH [8, 9] and a negative regulator of the Notch signaling [9, 10]. NRARP is expressed throughout human T cell development and its expression inversely correlates with NOTCH1 [11, 12]. In the mouse, overexpression of *Nrarp* in hematopoietic stem cells inhibits T-cell lineage commitment and early thymocyte development [12]. Our previous studies revealed that loss of *mir-181ab1* inhibits leukemia development at least in part by derepressing the expression of *Nrarp* [13]. These results suggested that deregulation of NRARP may contribute to the pathogenesis of T-ALL. Here, we uncover a dual role for NRARP dependent on NOTCH1 intracellular domain (NICD) levels, with opposite functional outcomes in T-ALL pathogenesis. Importantly, our findings establish a new paradigm in what regards the outcomes of the cross talk between Notch and Wnt signaling pathways in T-ALL, with important therapeutic implications.

## Results

### NRARP is upregulated in T-ALL cells but it is insufficient to block Notch signaling

**Fig. 1:**
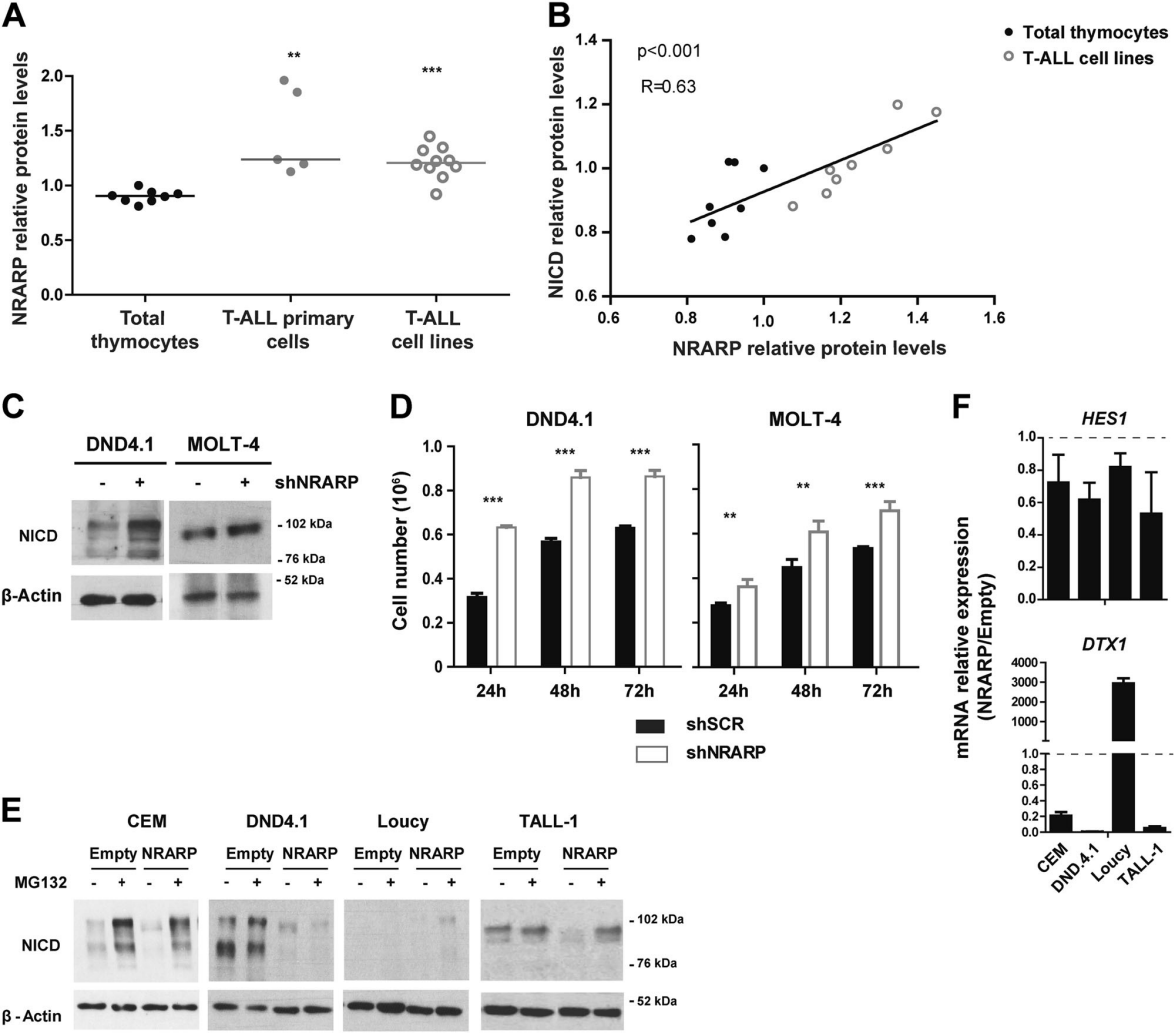
NRARP expression is increased in T-ALL cells but it is insufficient to block Notch signaling. **a** NRARP protein levels in T-ALL primary cells (*n* = 5) and cell lines (*n* = 10) determined by western blot (WB) and normalized to NRARP levels in total thymocytes (*n* = 8). **b** Correlation between NRARP and NICD protein levels in total thymocytes and T-ALL primary cell lines. Protein levels were determined by WB. **c** WB analysis of NICD expression in DND4.1 and MOLT-4 T-ALL cell lines upon *NRARP* knockdown using shRNAs. **d** Effects of *NRARP* knockdown in DND4.1 and MOLT-4 cell proliferation. Cells were transduced with an shRNA against *NRARP* (shNRARP) or a scramble sequence as control (shSCR). Representative assay of three biological experiments, each performed in triplicate. **e** Effects of *NRARP* overexpression in T-ALL cell lines NICD levels (determined by WB). To better visualize the changes induced by *NRARP* overexpression, T-ALL cells transduced with an Empty vector (control condition) or an NRARP vector were treated with the proteasome inhibitor MG132. **f** Relative expression of Notch transcriptional targets in T-ALL cells overexpressing *NRARP*. mRNA levels were normalized to control condition (Empty cells). In **d** and **f** data represent the mean ± SEM. Statistical values were obtained using either the Student’s *t* test (**a**), 2way ANOVA (**d**), or Pearson correlation (**b**). **p < 0.01, ***p < 0.001

To understand if NRARP plays a role in T-ALL pathogenesis we started by characterizing NRARP expression in T-ALL primary cells and cell lines. NRARP protein levels were upregulated in T-ALL cells in comparison with normal thymocytes (Fig. 1a). In addition, we observed a positive correlation between NRARP and NICD levels (Fig. 1b). These observations are consistent with the fact that NRARP is a direct transcriptional target of NOTCH1. Nonetheless, they also suggest that either the NRARP protein expressed in T-ALL cells is not functional or that its levels, although increased, are not sufficient to block NOTCH1 oncogenic signals. To address these questions, we used shRNAs to silence *NRARP* expression in the T-ALL cell lines DND4.1 and MOLT-4. Although we achieved a knockdown of only 40–50% at the mRNA level (Supplementary Fig. S1 A) that was sufficient to increase NICD levels in both cell lines (Fig. 1c). Consistent with the increase in NICD1 levels, DND4.1 and MOLT-4 cells knocked down for *NRARP* proliferated more than their parental counterparts (Fig. 1d). Rescue of *NRARP* expression in MOLT-4 shNRARP cells (Supplementary Fig. S1B) significantly decreased their proliferative capacity (Supplementary Fig. S1 C). Importantly, these results showed that NRARP is functional in T-ALL cells and that, as in normal T-cells, it negatively regulates the Notch pathway.

We next overexpressed *NRARP* in human T-ALL cell lines (Supplementary Fig. S1D, E), which led to NICD downregulation (Fig. 1e). NRARP has been shown to regulate NICD degradation through the proteasome [10]. Curiously, treatment of DND4.1 cells with the proteasome inhibitor MG132 did not reverse *NRARP* overexpression effects on NICD1 levels, suggesting that NRARP may also induce the degradation of NICD in a proteasome-independent way. Furthermore, *NRARP* overexpression blocked NOTCH transcriptional activity as shown by the overall decreased expression of NOTCH1 downstream targets *HES1* and *DTX1* in *NRARP*-overexpressing cells (Fig. 1f). Together, these results are consistent with the reported function of NRARP as a negative regulator of Notch signaling [9, 10], and suggest that in T-ALL NRARP levels, although elevated, are not sufficient to completely block NOTCH signals.

### NRARP can not only inhibit but also promote the proliferation of T-ALL cells

**Fig. 2:**
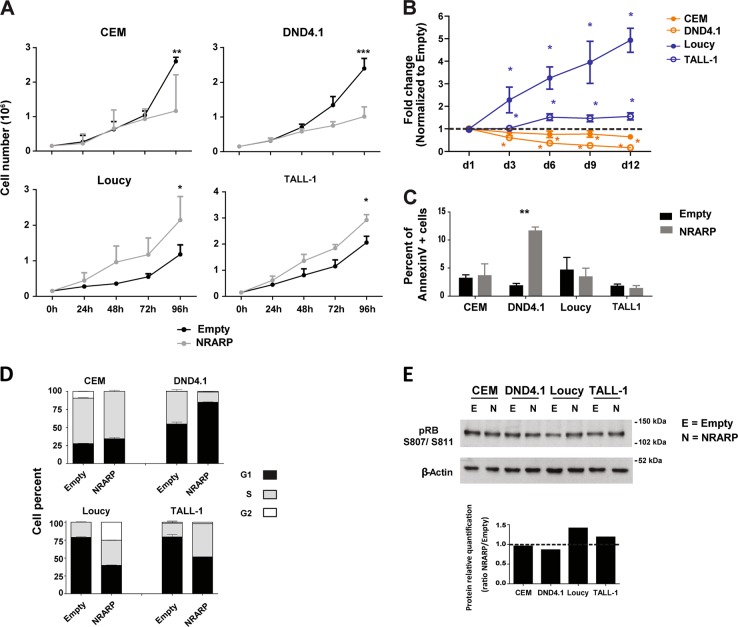
NRARP can not only inhibit but also promote the proliferation of T-ALL cells. **a** Effects of *NRARP* overexpression in T-ALL cell proliferation and **b** expansion. In **a** one of three independent assays (each performed in triplicate) is displayed, whereas in **b** the fold change induced by NRARP is shown in comparison to control (Empty condition). **c** Effects of *NRARP* overexpression in T-ALL cell death as determined by annexinV staining and flow cytometry analysis. One of three independent assays performed in triplicate is displayed. **d** Effects of *NRARP* overexpression in cell cycle progression. Cells were sorted in G1 and cultured for 8 h. *n* = 3. **e** Analysis of the protein levels of the cell cycle regulator pRB. In **a**, **b**, **c**, and **d** data represent the mean ± SEM. Statistical values were obtained using the Student’s *t* test. *p < 0.05, **p < 0.01, ***p < 0.001

Next, we investigated the functional effects of NRARP-induced inhibition of Notch signaling in T-ALL cell proliferation and survival. As expected, *NRARP* overexpression delayed proliferation and expansion of CEM and DND4.1 cell lines (Fig. 2a, b). However, to our surprise, NRARP had the opposite effect on Loucy and TALL-1 cells, promoting their proliferation and expansion (Fig. 2a, b).

With the exception of DND4.1 cells, we did not observe significant differences in leukemia cell viability upon *NRARP* overexpression (Fig. 2c). Thus, we next characterized NRARP effects on cell cycle progression. We sorted cells in G1 and analyzed cell cycle progression after 8 h in culture. Compatible with the proliferation data, we observed a delay in cell cycle progression in both CEM and DND4.1 *NRARP*-overexpressing cells (Fig. 2d). In contrast, Loucy and TALL-1 cells progressed faster in the cycle upon *NRARP* overexpression (Fig. 2d). In line with these results, we observed increased levels of phosphorylated retinoblastoma protein in *NRARP*-overexpressing Loucy and TALL-1 cells (Fig. 2e). When phosphorylated, this cell cycle regulator becomes inactivated, allowing for G1-to-S phase cell cycle transition.

### NRARP promotes Wnt signaling in T-ALL cells

**Fig. 3:**
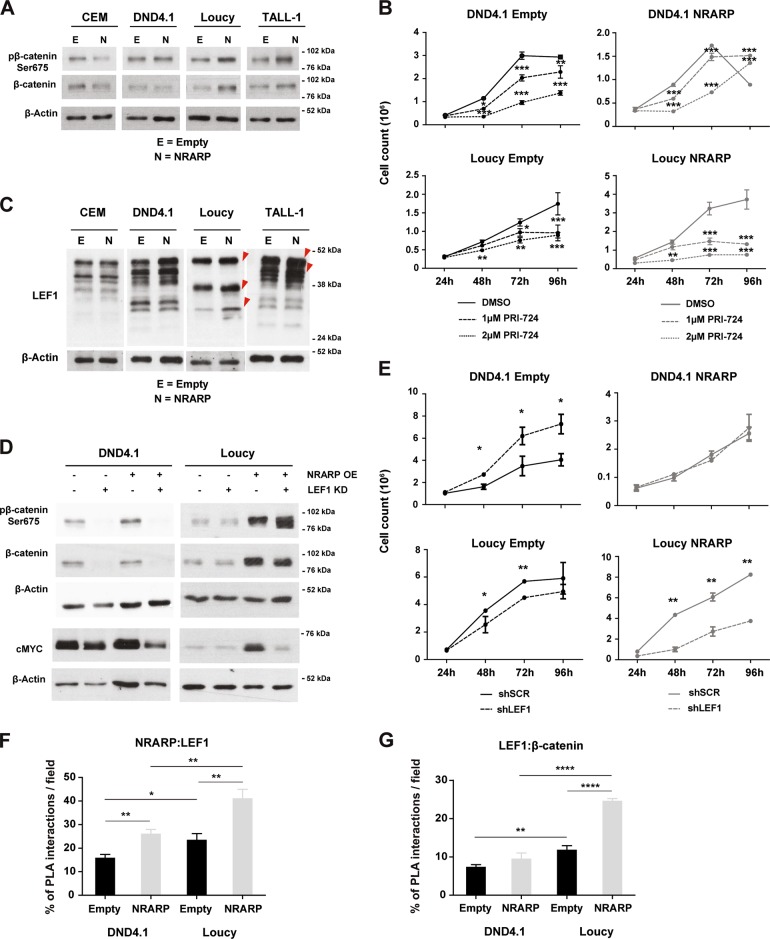
NRARP promotes Wnt signaling in T-ALL cells. **a** Analysis of WNT signaling activity by measuring β-catenin and pβ-catenin Ser675 protein levels by WB in T-ALL cell lines with and without *NRARP* overexpression. **b** Effects of Wnt inhibition in the proliferation of T-ALL cells overexpressing or not *NRARP* using the inhibitor PRI-724. One of three independent assays performed in triplicate is shown. **c** Analysis of LEF1 protein levels in T-ALL cell lines upon NRARP overexpression. LEF1 has several isoforms due to an alternative promoter and alternative splicing. **d** Effects of *LEF1* knockdown in β-catenin, pβ-catenin Ser675, and cMYC protein levels in T-ALL cell lines with and without NRARP overexpression. **e** Effects of LEF1 knockdown in the proliferation of T-ALL cells overexpressing or not *NRARP*. One of three independent assays performed in triplicate is shown. Quantitative analysis of **f** NRARP:LEF1 interactions and **g** LEF1-β-catenin interactions performed by the quantification of cells with and without PLA signals per cell per microscope field. The combination of three independent assays and the analysis of five microscope fields per assay are shown. In **b**, **e**, **f**, and **g** data represent the mean ± SEM. Statistical values were obtained using the Student’s *t* test. *p < 0.05, **p < 0.01, ***p < 0.001, ****p < 0.0001

Interestingly, we noticed that cMYC was not downregulated in Loucy and TALL-1 cells overexpressing *NRARP*. On the contrary *cMYC* transcript (Fig. S2A) and protein levels (Fig. S2B) were considerably upregulated in Loucy cells in particular. Since cMYC is a well-known transcriptional target of NOTCH, these data were in apparent contradiction with the inhibition of NOTCH we observed in the T-ALL cell lines, upon *NRARP* overexpression (Fig. 1f, e). On the other hand, these observations were consistent with the increased proliferation observed in Loucy and TALL-1 NRARP cells (Fig. 2) and suggested that NRARP may lead (directly or indirectly) to the activation of another pathway that maintains (or elevates) cMYC levels. Because in endothelial and neuro crest cells, NRARP has been shown to positively regulate canonical Wnt signaling [14, 15], which in turn it is known to regulate cMYC [16], we sought to investigate if NRARP could impact cMYC levels through the activation of the Wnt pathway. We started by evaluating β-catenin and phospho-β-catenin Ser675 levels as measures of Wnt signaling activity (phosphorylation of serine 675 has been shown to increase β-catenin stability and its transcriptional activity) [17] and found that both were upregulated in Loucy and TALL-1 cells overexpressing *NRARP* (Fig. 3a). These results suggested that *NRARP* overexpression positively regulates Wnt signaling in these cell lines. In contrast, *NRARP* overexpression in CEM and DND4.1 cells, did not affect or downregulated somewhat Wnt signaling pathway (Fig. 3a). Thus, to evaluate the impact of Wnt signaling in *NRARP*-overexpressing cells, we exposed T-ALL cells to the Wnt inhibitor PRI-724. This inhibitor binds to CPB blocking its interaction with β-catenin, inhibiting the transcription of Wnt downstream targets (Supplementary Fig. S3A) [18, 19]. Although Wnt inhibition initially affected the proliferation of both DND4.1 and Loucy NRARP cells, DND4.1 cells recovered their proliferative capacity while Loucy NRARP cells did not (Fig. 3b). Analysis of cell proliferation in parental cells further shows that Loucy NRARP cells are more sensitive to Wnt inhibition than Loucy Empty cells (Fig. 3b). Together, these results demonstrate that in Loucy cells overexpression of NRARP promotes proliferation through Wnt signaling. We also observed that Wnt inhibition decreased cell viability, in particular of Loucy NRARP cells (Fig. S3B). Curiously, blockade of Wnt signaling revert the loss in viability induced by NRARP in DND4.1 cells (Fig. 2c, Supplementary Fig. S3B), an effect that was partially maintained up to 144 h (Supplementary Fig. S3C). Supporting the importance of Wnt signaling pathway in the proliferative capacity of Loucy cells, we observed that activation of this pathway using a GSK3 inhibitor (Supplementary Fig. S3D) promotes Loucy Empty cells proliferation (Supplementary Fig. S3E). The activation of this pathway by NRARP in Loucy cells may explain the fact that, in Loucy NRARP cells, treatment with GSK3 inhibitor does not promote proliferation (Supplementary Fig. S3E). In what concerns DND4.1 Empty and NRARP cells we observed a decrease in proliferation, which is consistent with the role described for GSK3 in the positive regulation of Notch1 signaling and NICD1 stability (Supplementary Fig, S3E) [20]. As shown in the Supplementary Fig. S3D treatment with the GSK3 inhibitor leads to a downregulation of NICD1 levels.

Of note, while NRARP negatively modulates Notch signaling by promoting NICD degradation, it is known to positively regulate Wnt signaling by promoting LEF1 protein stability [14]. LEF1 is a DNA-binding transcription factor of the T-cell factor (TCF) family, acting downstream of the Wnt signaling pathway by interacting with nuclear β-catenin [21]. Analysis of LEF1 protein levels in *NRARP*-overexpressing cells showed an increase in Loucy and TALL-1 cells of 27% and 13%, respectively (Fig. 3c). LEF1 has several isoforms with apparently different, sometimes opposing, functions [22–24]. Thus, we next dissected the impact of LEF1 downstream from NRARP by silencing LEF1 in DND4.1 and Loucy cells with and without *NRARP* overexpression (Supplementary Fig. S4A, B). LEF1 knockdown blocked Wnt signaling in both Empty and NRARP cells (Fig. 3d). We then evaluated the functional effects of LEF1 knockdown in NRARP expressing cells and found that LEF1 silencing reversed NRARP-induced proliferation in Loucy cells, whereas it did not impact DND4.1 NRARP cells (Fig. 3e). These changes in Loucy NRARP cells were paralleled by cMYC downregulation (Fig. 3d). This set of results provide evidence that NRARP, aside from having a suppressive role in T-ALL by inhibiting Notch signaling (e.g., in CEM and DND4.1 cells) can also promote T-ALL proliferation by potentiating Wnt signaling through LEF1 (e.g., in Loucy and TALL-1). Moreover, the fact that LEF1 knockdown in DND4.1 control cells augmented cell proliferation, suggests that Wnt signaling may has an inhibitory effect in some T-ALL cases (Fig. 3e). This inhibitory effect of LEF1 knockdown was further confirmed in the MOLT-4 cells (Supplementary Fig. S4C, D). In both cell lines, although not statistically significant, LEF1 knockdown results in minor increments in NOTCH1 downstream targets (Supplementary Fig. S4E).

To understand if NRARP regulates LEF1 in a direct fashion we carried out in situ PLA assays to evaluate the interaction between NRARP and LEF1 (Supplementary Fig. S5A). We found that, in both DND4.1 and Loucy cell lines, *NRARP* overexpression increased the percentage of cells with NRARP:LEF1 interactions (Fig. 3f) and, in the case of Loucy cells, the number of interactions per cell (Supplementary Fig. S5B). However, this increase in NRARP:LEF1 interactions only translated into increased Wnt signaling activity in Loucy cells as determined by the increase in LEF1:β-catenin interactions in these cells upon *NRARP* overexpression (Fig. 3g and Supplementary Fig. S5 C, D). These data are consistent with the functional results obtained by the chemical inhibition of Wnt signaling and LEF1 knockdown, and show that although NRARP interacts with LEF1 in both cell lines, it leads to Wnt signaling activity only in Loucy cells.

### NRARP anti- or pro-tumoral role in T-ALL depends on NICD1 levels

**Fig. 4:**
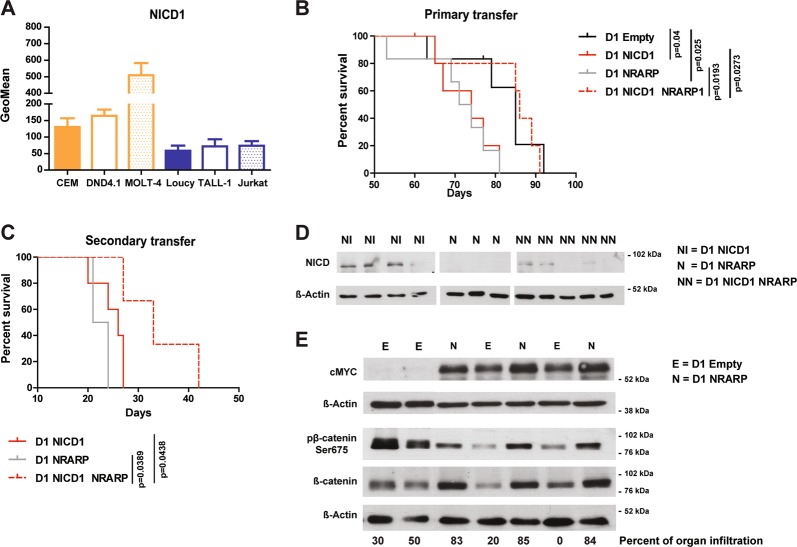
NRARP anti- or pro-tumoral role in T-ALL depends on NICD1 levels. **a** Flow cytometry analysis of NICD1 levels in T-ALL cell lines (*n* = 3). **b** Kaplan–Meier survival curve of immunocompromised (NSG) mice transplanted with p53-null CD4-CD8- precursor T-cells (D1 cells) overexpressing *NICD1*, *NRARP* or both. Whereas NRARP alone induced leukemia development with a similar kinetic of that of NICD1 (median survival of 72.5 and 74 days, respectively), its co-expression with NICD1 had a suppressive effect, increasing significantly the median survival to 86 days (*p* < 0.05). **c** Kaplan–Meier survival curve of NSG mice transplanted with D1 cells isolated from mice primarily transplanted with D1 cells overexpressing *NICD1*, *NRARP*, or both. The secondary transfer of these cells confirmed their leukemogenic potential and NRARP suppressive role when co-expressed with NICD1 (*p* < 0.05). **d** NICD protein levels in D1 cells isolated from spleens of mice transplanted with D1 NICD1, D1 NRARP, or D1 NICD1 *NRARP*-overexpressing cells. **e** Western blot analysis of cMYC, β-catenin, and pβ-catenin Ser675 protein levels in cells collected from spleens of mice transplanted D1 NRARP cells and D1 Empty cells (control condition)

CEM and DND4.1 cells have *NOTCH1*-activating mutations while Loucy and TALL-1 cells do not [25] (of note TALL-1 cells have a *NOTCH3* mutation [26]). Consequently, CEM and DND4.1 cells have higher levels of *NICD1* than Loucy and TALL-1 cells (Fig. 4a). Therefore, we investigated whether the dual role of NRARP related to NICD1 levels. First we confirmed that the dual effect of NRARP correlated with NICD1 levels also in MOLT-4 and Jurkat T-ALL cells (Supplementary Fig. S1F, G). Although these cell lines have *NOTCH1*-activating mutations [25], they have very different levels of NICD1 (Fig. 4a). Taken together, our data suggest that NRARP may prevent the expansion of cells with high levels of NICD1 (such as the *NOTCH1*-mutant cell lines CEM, DND4.1, and MOLT-4) and promote the growth of cells with normal (lower) levels of NICD1 (e.g. Loucy, TALL-1, and Jurkat cell lines).

To further test this possibility we overexpressed NICD1 in the p53-null CD4−CD8− precursor T-cell line D1 [27], with or without co-expression of *NRARP* (Supplementary Fig. S6). D1 cells were then transplanted into immunocompromised mice. As expected, mice transplanted with NICD1-expressing D1 cells developed leukemia rapidly, whereas mice transferred with D1 cells co-expressing NICD1 and NRARP developed leukemia significantly later (Fig. 4b). By contrast, mice transplanted with D1 cells overexpressing *NRARP* alone developed leukemia with similar kinetics to those transplanted with D1 NICD1 cells (Fig. 4b), confirming the oncogenic potential of NRARP in the context of low levels of Notch1 signaling. Transfer of these cells into secondary recipient mice confirmed their leukemogenic nature (Fig. 4c). In agreement with the in vitro data using human T-ALL cell lines, NRARP NICD1 D1 cells, collected from the spleen, showed decreased NICD1 levels as compared with D1 overexpressing NICD1 alone (Fig. 4d). Moreover, D1 cells expressing NRARP alone displayed higher levels of β-catenin and cMYC than control, empty vector cells (Fig. 4e).

### NRARP negative impact on primary T-ALL cells associates with Notch pathway activation status

**Fig. 5:**
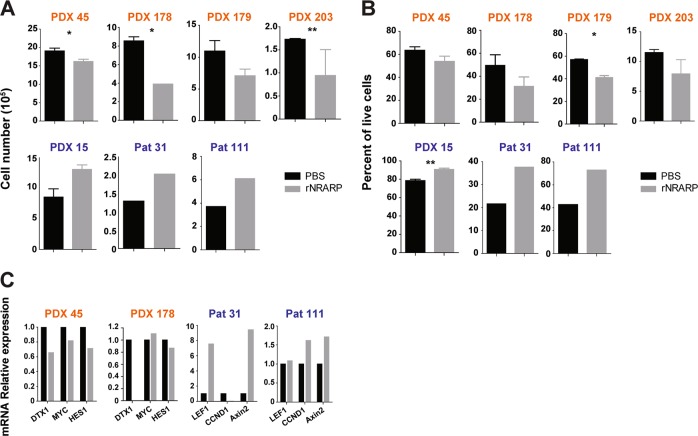
NRARP negative impact on primary T-ALL cells associates with Notch pathway activation status. Analysis of primary and patient-derived xenograft (PDX) T-ALL cell proliferation **a** and **b** viability upon delivery of NRARP recombinant protein (rNRARP). Samples with *NOTCH1* mutations/high levels of NICD1 are depicted in orange and samples *NOTCH1-*WT/low levels of NICD1 in blue. **c** mRNA analysis of Notch1 and Wnt signaling transcriptional targets by quantitative-PCR, in primary and PDX T-ALL cells treated with rNRARP. In **a** and **b** data represent the mean ± SEM. Statistical values were obtained using the Student’s *t* test. *p < 0.05, **p < 0.01

To validate the dual role of NRARP in human primary T-ALL cells we exposed primary or patient-derived xenograft (PDX) T-ALL cells, classified according to their *NOTCH1* mutational status/levels of NICD1 (Supplementary Fig. S7A, B) [28], to NRARP recombinant protein (rNRARP) (Supplementary Fig. S7C, D). Consistent with our observations in cell lines, NRARP forced expression in *NOTCH1* mutant/NICD1 high T-ALL samples negatively impacted cell number (Fig. 5a) and viability (Fig. 5b), whereas it had the opposite effect on *NOTCH1* wild type/NICD low cases (Fig. 5a, b). Also in agreement with our observations in T-ALL cell lines, rNRARP led to the downregulation of NOTCH targets in *NOTCH1*-mutant samples and upregulated WNT targets in *NOTCH1*-WT cells (Fig. 5c). Overall, the data from primary T-ALL samples and PDXs support our hypothesis that NRARP plays a dual role in T-ALL, with opposite functional outcomes that depend on NICD1 levels.

## Discussion

**Fig. 6:**
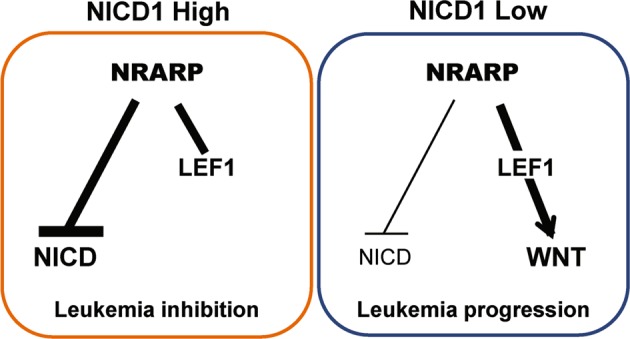
Schematic representation of NRARP dual role in T-ALL. In cells with high levels of NICD1, NRARP overexpression blocks Notch signaling and suppresses leukemia development. In cells with low/normal levels of NICD1, NRARP activates the Wnt pathway promoting leukemia progression

*NRARP* is a transcriptional target of NOTCH1 that promotes the degradation of NICD, originating a negative feedback loop that downregulates Notch signaling. Although it is known that the constitutive activation of this signaling pathway plays a pivotal role in T-ALL, a putative role for NRARP in this disease context has not been investigated. Our studies on the role of *mir-181ab1* in T-ALL suggested that NRARP could have a suppressive role in T-ALL [13]. Thus, in the current work we set out to evaluate the contribution of NRARP to T-ALL pathogenesis. Interestingly, our work demonstrates that NRARP has a dual role in T-ALL, dependent of Notch1 activity levels. On one hand, NRARP has a ‘tumor suppressor’-like role in T-ALL cells with high levels of NICD by inhibiting Notch1 signaling. On the other hand, in T-ALL cells with normal levels of NICD, NRARP unexpectedly promotes leukemogenesis, by activating the Wnt signaling pathway (Fig. 6).

NRARP is expressed in hematopoietic progenitor cells [11], as well as in thymocyte subsets, with highest levels in CD4+CD8+ T-cells [12]. Its overexpression in murine bone marrow stem/progenitor cells blocks T-cell lineage commitment and the progression of T-cell development through DN stages [12]. Notably, we now show that NRARP overexpression leads to the malignant transformation of CD4−CD8− T-cell precursors, inducing T-ALL as efficiently as NOTCH1 but through Wnt signaling activation. Our work identifies an unexpected role for NRARP in T-ALL pathogenesis and warrants the search for genomic lesions (or any other alteration—–e.g., transcriptional, epigenetic, or post transcriptional) that may lead to the deregulation of NRARP levels and/or function. According to our findings, an alteration leading to the upregulation of NRARP levels in NOTCH WT cells can promote leukemogenesis through the activation of the Wnt signaling. On the opposite, alterations that lead to the downregulation of NRARP levels in a NOTCH1 high context, can promote leukemogenesis by derepressing Notch oncogenic signaling. Thus, if identified, it would be interesting to understand how these alterations correlate with the activation of Notch and Wnt pathways.

Importantly, our results highlight the therapeutic potential of NRARP in a context of high levels of NOTCH or NOTCH1 mutations. We show that, although increased in T-ALL cells with higher levels of NOTCH, NRARP is not sufficient to block those oncogenic signals. However, delivery of NRARP recombinant protein to T-ALL cells with high levels of NOTCH negatively impacts their proliferation and viability, as demonstrated by our studies. This implicates that therapeutic strategies leading to NRARP activation will be potentially useful for the treatment of a majority of T-ALL cases (with NOTCH1 hyper activation). Moreover, our findings also demonstrate that such strategies may be detrimental in the context of some *NOTCH1-*WT/low NICD1 T-ALL cases and/or Wnt signaling dependent T-ALL cases.

NRARP has been shown to independently regulate canonical Wnt and Notch signaling [14, 15]. Curiously, NRARP negatively modulates the Notch pathway by promoting NICD degradation, but positively regulates Wnt signaling by promoting LEF1 protein stability [14]. LEF1 is a DNA-binding transcription factor of the TCF family, acting downstream of the Wnt signaling pathway by interacting with nuclear β-catenin [21]. Our data demonstrates that in T-ALL, NRARP also positively regulates Wnt signaling through LEF1. Overexpression of *NRARP* increases LEF1 protein levels and knockdown of LEF1 reverses NRARP-induced proliferation in T-ALL cells with low levels of NICD1. In addition, our data shows that NRARP overexpression increases the number of NRARP:LEF1 interactions which in T-ALL cells with low levels of NICD1 translates into more LEF1:β-catenin interactions. Because NRARP binds to NICD1 and LEF1, it is possible that in cells with high levels of NICD1, by targeting NICD to degradation, NRARP drags LEF1 along, impairing Wnt signaling activation.

Given their essential roles throughout T-cell development, the cross talk between Notch and Wnt pathways has been postulated to also occur in T-ALL. Nonetheless, this has not been clearly demonstrated and the existing data are not consensual. Some studies point for a positive correlation between these pathways. For example, in adult T-ALL, *LEF1* activating mutations were associated with *NOTCH1* mutations [29]. In addition, *Lef1* has been reported as a transcriptional target of NOTCH1 in T-cell lymphomas and to accelerate *Notch1*-induced lymphomagenesis in mice [30]. In contrast, other evidences point to the inactivation of Wnt signaling when Notch signaling is active [31–33]. Particularly, in pediatric T-ALL, *LEF1* inactivation has been associated with *NOTCH1*-activating mutations [34]. The fact that proliferation is potentiated by the chemical inhibition of Wnt signaling and by LEF1 silencing in *NOTCH1*-mutant DND4.1 and MOLT-4 cells is in agreement with the latter studies, and suggests that Wnt signaling may be detrimental for T-ALL cells with *NOTCH1* constitutive activation.

Overall, our results identify a role for NRARP in T-cell leukemogenesis and indicate that Wnt signaling has an oncogenic role in human T-ALL pathogenesis, independent of Notch signals. Our findings open new therapeutic perspectives and underline the need to consider *NOTCH1* and *WNT* mutational status into T-ALL therapeutic decisions. While the therapeutic inhibition of Notch signaling may be beneficial in patients with the constitutive activation of this pathway, in patients with low levels of Notch signaling this approach may be therapeutically disadvantageous. In these cases, the inhibition of Wnt signaling may prove to be beneficial. In contrast, our data further suggests that Wnt signaling inhibition may be disadvantageous for T-ALL patients with high levels of Notch1 activity. Importantly, our findings may extend to other cancer types where Notch and Wnt are known to play a role, such as breast, lung, and colon cancer.

## Materials and methods

### Primary leukemia cells

Primary leukemia cells were obtained from bone marrow and/or peripheral blood of diagnostic pediatric T-ALL. Informed consent was obtained in accordance with the Declaration of Helsinki and under the ethical review board approval of Instituto Português de Oncologia (Lisbon, Portugal) and Centro Infantil Boldrini (Campinas, São Paulo, Brazil). When available, T-ALL cells from PDXs were used.

### Cell lines

T-ALL cell lines CEM, MOLT-4, DND4.1, Jurkat, Loucy, and TALL-1 were cultured in RPMI 1640 (Gibco, Thermo Fisher Scientific, Gaithersburg, MD, USA) supplemented with 10% FBS (Biowest, Nuaillé, France), 1% penicillin/streptomycin (Gibco) and 1% HEPES (Gibco), at 37 °C in a 5% CO_2_ environment. Cells were kept at an optimal concentration of 0.5 × 10^6^ cells/mL. D1 cells were cultured in the same conditions plus 25 ng/mL rmIL-7 (PeproTech EC, London, UK). HEK-293T cells were maintained in DMEM (Gibco) supplemented with 10% FBS, 1% l-glutamine, and 1% penicillin/streptomycin. All cell lines were periodically checked for mycoplasma by PCR and were found to be negative.

### RNA extraction, cDNA synthesis, and quantitative-PCR

Total RNA was extracted using TRIzol® reagent (Ambion™, Thermo Fisher Scientific). Synthesis of cDNA was performed with up to 1 μg of total RNA, using the reverse transcriptase SuperScript™ III kit (Invitrogen, Thermo Fisher Scientific) and random hexamers. Quantitative-PCR was performed using Taqman and SyberGreen methods. *NRARP* and *18S* expression was quantified using Taqman Gene Expression Assays (Applied Biosystems, Foster City, CA, USA). The expression of the remaining genes was quantified using Power SYBR Green PCR Master Mix (Applied Biosystems) and the specific primers are indicated in Supplementary Table S1. The PCR reactions were performed using a ViiA™ 7 Real-Time PCR System (Thermo Fisher Scientific). The expression of each gene was normalized to the expression level of the ribosomal RNA *18S* using the dCt method and fold difference comparisons between samples evaluated using the ddCT method.

### Immunoblot

Lysates were prepared as described [35], resolved by SDS-PAGE and immunoblotted with antibodies against NRARP (E-13 and C-12), β-Catenin (E-5), β-Actin (Santa Cruz Biotechnology, Dallas, TX, USA), phospho-β-catenin (Ser675), LEF1 (C18A7), Cleaved Notch1 (Val1744), cMyc (D3N8F) (Cell Signaling Technology, Danvers, MA, USA), Rb (G3-245), and phospho-Rb (S807/S811, J112-906) (BD Pharmingen^TM^, San Jose, CA, USA). Densitometry analysis was performed using Adobe Photoshop CS5 Extended software (Adobe Systems). Results were normalized to the loading control.

### Proximity ligation assay (PLA)

We utilized the Duolink® PLA technology for the in situ detection of NRARP:LEF1 and LEF1:β- catenin interaction in T-ALL cell lines with and without NRARP overexpression. We used the Duolink® In Situ Red Starter Kit Goat/Rabbit and In Situ Red Starter Kit Mouse/Rabbit (Sigma-Aldrich, St. Louis, MO, USA) for the detection of NRARP:LEF1 and LEF1:β-catenin interactions, respectively. We followed manufacturer’s instructions and used the primary antibodies anti-LEF1 Rabbit mAb (HPA002087) (Sigma-Aldrich), anti-β-catenin mouse mAb (E-5), and anti-NRARP goat mAb (E-13) (Santa Cruz Biotechnology). Samples were analyzed in a confocal laser point-scanning microscope (Zeiss LSM 710), using the integrated software ZEN 2012. Cell counting and image configuration were performed using Fiji/ImageJ software.

### Proteasome inhibitor treatment

Cells were treated with 1 µL/mL of MG132 inhibitor (10 mM in DMSO) (Calbiochem), at a cell density of 1 × 10^6^ cells/mL and incubated for 8 h at 37 °C. Cells were then harvested and lysed as described above.

### Production of retro and lentiviruses

Retroviruses were produced using the pCLeco packaging vector and vesicular-stomatitis-virus-pseudotyped lentiviruses using a third-generation system. Both viruses were produced by co-transfection into HEK-293T cells and using Lipofectamine 2000 (Life technologies, Carlsbad, CA, USA) as previously describe [13, 35].

### Transduction of cell lines for gene overexpression or knockdown

*NRARP*-overexpressing cells (referred to as NRARP) were established by transduction with lentiviruses produced using the lentiviral vector pCDH-CMV-MCS-T2AcopGFP (SBI, Palo Alto, CA, USA) cloned with NRARP ORF [13]. Cells transduced with the pCDH vector without an insert are referred to as “Empty”. For NICD overexpression, cells were transduced with retroviruses produced using the retroviral construct MigR1-NICD1 [13]. Cell lines knocked down for NRARP or LEF1 was established using OmicsLink™ shRNA vectors from GeneCopoeia (Rockville, MD, USA). The control vector (named SCR) expresses a scramble shRNA sequence. Briefly, 1 × 10^6^ cells were incubated with 1 mL of viral supernatant and 8 ng/mL of polybrene (Sigma-Aldrich). Cells were spun down for 120 min at room temperature at 2300 rpm. The resulting transduced cell lines were sorted for an equivalent reporter expression.

### Flow cytometry analysis

Standard procedures were used to stain the cells with fluorochrome conjugated antibodies or to verify reporter protein expression. In this study we used a PE anti-NOTCH1 (mN1A) antibody (Biolegend, San Diego, CA, USA). Samples were acquired in an LSRFortessa cell analyzer (BD Biosciences). Data analyses were performed using the FlowJo software.

### Cell growth assays

To assess cell growth potential of T-ALL cell lines, expansion and proliferation assays were carried out. Expansion assays were performed by culturing a fixed number of cells at optimal density and counting cells in a hemocytometer using trypan blue for dead cells exclusion, at days 3, 6, 9, and 12 of culture. For proliferation experiments 1.5 × 10^5^ cells were plated at optimal density and harvested at 24, 48, 72, and 96 h. Cell number was determined by flow cytometry (LSRFortessa cell analyzer, BD Biosciences) using counting beads (Beckmon Coulter). Data analyses were performed using FlowJo™ software.

### Viability assays

To assess cell viability 1.5 × 10^5^ cells were plated at optimal density and harvested at 24, 48, 72 and 96 h. Viability was determined by flow cytometry (LSRFortessa cell analyzer, BD Biosciences) using annexinV-APC (eBioScience, Thermo Fisher Scientific) and 7AAD dye (BD Pharmingen), according to manufacturer’s protocol. Data were analyzed using FlowJo™ software.

### Cell cycle analysis

To analyze cell cycle profiles of *NRARP*-overexpressing T-ALL cell lines (as comparing with empty control vectors), 1 × 10^6^ cells were incubated for 1 h at 4 °C and plated afterwards into 12-well plates at optimal density.

To determine the effects of NRARP in T-ALL cell cycle progression we sorted T-ALL cells in the G1 phase of cell cycle and compared the cell cycle profile of those cells upon 8 h in culture. We used the Vybrant® DyeCycle™ Violet Stain dye (Thermo Fisher Scientific) to sort cells in G1 phase using a BD FACSAria™ Cell Sorter (containing a violet laser, 407 nm). Briefly, for cell cycle analysis, cells were collected, washed, resuspended in cold PBS, and fixed with equal volume of 80% ethanol in PBS. Before staining, cells were washed twice with cold PBS and incubated with RNase A solution of 50 µg/mL in PBS, for 30 min at 37 °C. Propidium iodide was added at a concentration of 2.5 × 10^−3^ µg/µL and incubated overnight. Cell cycle profile was assessed next day by flow cytometry using a FACSCalibur. Data analyses were performed using FlowJo™ or ModFit LT™ software, as appropriate.

### NRARP recombinant protein production

Plasmid encoding recombinant NRARP protein was used to transform BL21 bacteria to facilitate isopropyl-b-D-thiogalactoside induction for protein expression in LB medium cultures following standard methods. Eluents containing protein were measured using Bradford reagent, combined and dialyzed overnight at 4 °C and stored at appropriate temperature until further use.

### D1 cells transfer in vivo model

Age and sex-matched (8–14 weeks) NSG mice were transferred with D1 cells overexpressing NRARP and/or NICD not blinding. Cells (5 × 10^6^) were injected intravenously in the tail. For the analysis of mouse overall survival humane endpoints were established. In particular, mice were euthanized when presenting a 20% weight loss or signs of lethargy. These endpoints were used to build the survival curve. The Kaplan–Meier estimator was used to determine the median rate of survival. The *p*-value was determined using the log-rank (Mantel–Cox) test. At the time of the humane endpoints mice were sacrificed with anesthesia overdose (Isoflurane). Experimental procedures were approved by the institutional Animal Ethics Committee from Instituto de Medicina Molecular João Lobo Antunes and followed the recommendations for the care and use of laboratory animals from the European Commission and Portuguese authorities. The minimal number of animals sufficient for the generation of statistically significant data (*n* = 5) was used.

### Statistical analysis

GraphPad Prism software was used for statistical analysis. Unless stated otherwise functional assays were evaluated using the unpaired Student’s two-tailed *t*-test. Significance was set for *p* < 0.05 (*p < 0.05; **p < 0.01; ***p < 0.001: ****p < 0.001).

## Supplementary information

Table S1

Supplementary Figure Legends

Supplementary Figure S1

Supplementary Figure S2

Supplementary Figure S3

Supplementary Figure S4

Supplementary Figure S5

Supplementary Figure S6

Supplementary Figure S7
