# Supplementary material for: NRARP displays either pro- or anti-tumoral roles in T-cell acute lymphoblastic leukemia depending on Notch and Wnt signaling
Source: Oncogene. 2019 Oct 4;39(5):975–86. doi: 10.1038/s41388-019-1042-9 (PMC6989401; doi:10.1038/s41388-019-1042-9)
Supplement: Supplementary file 1 — Table S1 [file 41388_2019_1042_MOESM1_ESM.doc]

| **Gene** | **Forward primer** | **Reverse primer** |
| --- | --- | --- |
| h*DTX1* | CAG CTT GTG CCC TAC ATC ATC | ACG ACG GGT CGT AGA AGT TG |
| h*HES1* | GCA GAT GAC GGC TGC GCT GA | AAG CGG GTC ACC TCG TTC ATG C |
| h*MYC* | GGC TCC TGG CAA AAG GTC A | CTG CGT AGT TGT GCT GAT GT |
| h*NOTCH1* | GAG GCG TGG CAG ACT ATG C | CTT GTA CTC CGT CAG CGT GA |
| h*CCND1* | CAA TGA CCC CGC ACG ATT TC | CAT GGA GGG CGG ATT GGA A |
| hLEF1 | TGC CAA ATA TGA ATA ACG ACC CA | GAG AAA AGT GCT CGT CAC TGT |
| h*AXIN2* | CTC CCC ACC TTG AAT GAA GA | GTT TCC GTG GAC CTC ACA CT |
| h*18S* | GGA GAG GGA GCC TGA GAA ACG | CGC GGC TGC TGG CAC CAG ACT T |

**Table S1.** List of primers used in quantitative-PCR.
