# Supplementary material for: NRARP displays either pro- or anti-tumoral roles in T-cell acute lymphoblastic leukemia depending on Notch and Wnt signaling
Source: Oncogene. 2019 Oct 4;39(5):975–86. doi: 10.1038/s41388-019-1042-9 (PMC6989401; doi:10.1038/s41388-019-1042-9)
Supplement: Supplementary file 2 — Supplementary Figure Legends [file 41388_2019_1042_MOESM2_ESM.docx]

**Supplementary Figure Legends:**

**Figure S1: Modulation of NRARP expression in T-ALL cell lines.** (A) Quantification of *NRARP* mRNA by quantitative PCR in DND4.1 and MOLT-4 cells transduced with shRNAs against *NRARP* (shNRARP) or a scramble sequence (shSCR). Expression in shNRARP cells was lower than in shSCR cells (n=3). (B) Analysis of NRARP expression in MOLT-4 cells knocked-down for *NRARP* transduced with an Empty or *NRARP*-rescue expression vector by quantitative PCR. *NRARP rescued* expressing cells had upregulated levels of NRARP. *NRARP* mRNA expression was normalized to the control condition (ShSCR_Empty vector condition). (C) Effects of *NRARP* rescued expression in MOLT-4 cells proliferation. (D and E) Analysis of NRARP expression in T-ALL cell lines transduced with a *NRARP* overexpression vector by (D) quantitative PCR and (E) western blot. *NRARP*-overexpressing cells had upregulated levels of NRARP. (D) *NRARP* mRNA expression was normalized to the control condition (Empty vector). (E) Cells were treated with the proteasome inhibitor MG132 to better discriminate the differences in NRARP at protein level. (F) Analysis of *NRARP* mRNA expression by quantitative PCR in MOLT-4 and Jurkat cell lines after *NRARP* overexpression. *NRARP* expression was upregulated in cells transduced with the NRARP overexpression vector in comparison with cells transduced with the empty vector. (G) Effects of *NRARP* overexpression in T-ALL cells proliferation. One of three independent assays (each performed in triplicate) is depicted. In (A), (C) and (G) data represent the mean ± SEM. Statistical values were obtained using the Student’s t test. *p<0.05, **p<0.01.

**Figure S2: Effects of NRARP overexpression in cMYC levels.** (A) Relative expression of *cMYC* transcriptional targets in T-ALL cells overexpressing *NRARP*. mRNA levels were normalized to control condition (Empty cells). (B) Western blot analysis of cMYC levels in control and NRARP overexpressing cells. In (A) data represent the mean ± SEM.

**Figure S3: Chemical modulation of Wnt signaling in T-ALL cell lines.** To verify if T-ALL cells were sensitive to Wnt inhibition, cells were treated with the Wnt inhibitor PRI-724. (A) Quantitative analysis by PCR of Wnt transcriptional targets in T-ALL cells exposed to different concentrations of PRI-724 showed that 1µM is sufficient to impair Wnt transcriptional activity. (B) Flow cytometry analysis of DND4.1 and Loucy cells viability upon treatment with 1µM and 2µM of PRI-724. One of three independent assays performed in triplicate is shown. (C) Flow cytometry analysis of DND4.1 viability at 144h upon treatment with 1µM and 2µM of PRI-724. One of two independent assays performed in triplicate is shown. To verify if T-ALL cells were sensitive to Wnt activation, cells were treated with the GSK3 inhibitor CHIR9901. (D) Western blot analysis of β-catenin, Wnt transcriptional targets and NICD1 in T-ALL cells exposed to different concentrations of CHIR9901 showed that 3µM lead to β-catenin accumulation and Wnt signaling activation. (E) Effects of CHIR9901 in T-ALL cells proliferation. One of three independent assays (each performed in triplicate) is depicted. In (A), (B), (C) and (E) data represent the mean ± SEM. Statistical values were obtained using the Student’s t test. *p<0.05, **p<0.01, ***p<0.001.

**Figure S4: Modulation of LEF1 expression in T-ALL cell lines.** Analysis of LEF1 expression in T-ALL cells after *LEF1* knockdown by (A) quantitative PCR and (B) western blot. Cells transduced with shRNAs against *LEF1* expressed lower levels of LEF1 than cells transduced with a scramble shRNA (shSCR). (C) Analysis of LEF1 expression in MOLT-4 cells after *LEF1* knockdown by quantitative PCR. MOLT-4 cells transduced with shRNA against *LEF1* expressed lower levels of LEF1 than cells transduced with a scramble shRNA (shSCR). (A and C) *LEF1* mRNA expression was normalized to the shSCR condition. (D) Effects of LEF1 knockdown in the proliferation of MOLT-4 cells. One of two independent assays performed in triplicate is shown. (E) Effects of LEF1 knockdown in NOTCH1 downstream targets by quantitative PCR. In (C), (D) and (E) data represent the mean ± SEM. Statistical values were obtained using the Student’s t test. *p<0.05, **p<0.01.

**Figure S5: NRARP interacts with LEF1 and modulates LEF1 interactions with β-catenin.** Analysis of NRARP and LEF1 (A) and LEF1 and β-catenin (B) co-localization by *in situ* PLA in DND4.1 Empty and NRARP cells and in Loucy Empty and NRARP cells. Red fluorescent dots indicate interaction spots (PLA signals) and blue indicates cell nuclei. The negative control were performed in the absence primary antibodies. The quantitative analysis of (B) NRARP:LEF1 interactions and (D) LEF1-β-catenin interactions was performed by the quantification of PLA signals per cell per microscope field. (B) and (D) depict the data of three independent assays and the analysis of 5 microscope fields per assay. Statistical values were obtained using the Student’s t test. *p<0.05, ****p<0.0001.

**Figure S6: Overexpression of NRARP and / or NICD1 in D1 T-cell line.** The p53-null CD4-CD8- precursor T-cell line D1 was transfected with *NICD1* and/or transduced with *NRARP*. Overexpression efficiency was confirmed at the protein level by WB (NRARP) or flow cytometry (NICD).

**Figure S7: Delivery of NRARP recombinant (rNRARP) protein to patient and patient derived xenograft T-ALL cells.** (A) Analysis of NICD protein levels in primary and patient derived xenograft (PDX) T-ALL samples, by western blot. (B) Information regarding *NOTCH1* mutational status determined by DNA sequencing (24). Samples with *NOTCH1* mutations/high levels of NICD are depicted in orange and samples *NOTCH1*-WT/low levels of NICD1 in blue. N.D. not determined. (C) Schematic representation of the construct used to produce NRARP recombinant protein. (D) Analysis of NRARP levels in primary and patient derived xenograft cells treated with NRARP recombinant protein (2.5µM).
