# Supplementary figures and images for: NRARP displays either pro- or anti-tumoral roles in T-cell acute lymphoblastic leukemia depending on Notch and Wnt signaling
Source: Oncogene. 2019 Oct 4;39(5):975–86. doi: 10.1038/s41388-019-1042-9 (PMC6989401; doi:10.1038/s41388-019-1042-9)

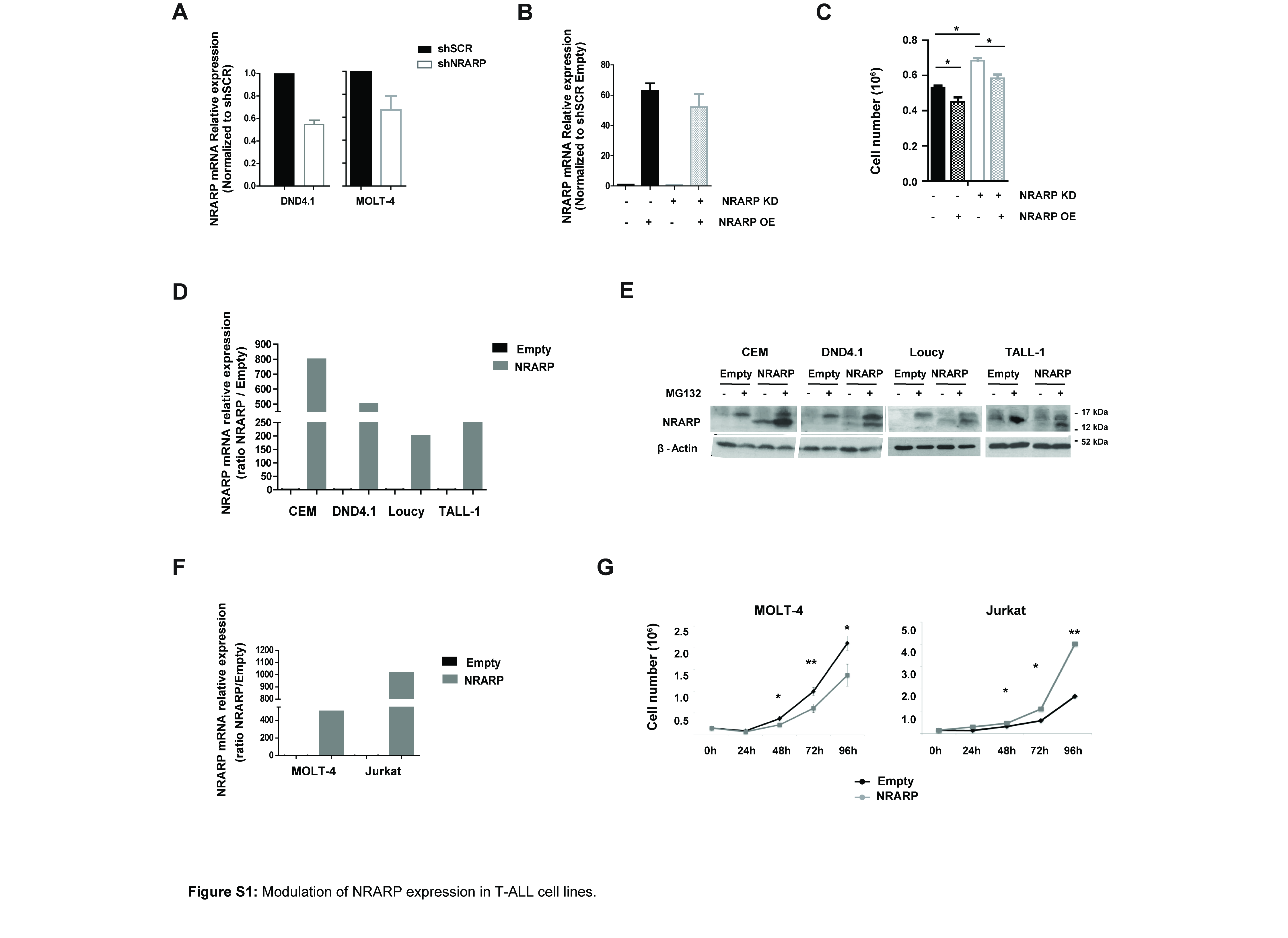

Supplement: Supplementary file 3 — Supplementary Figure S1 [file 41388_2019_1042_MOESM3_ESM.tif]

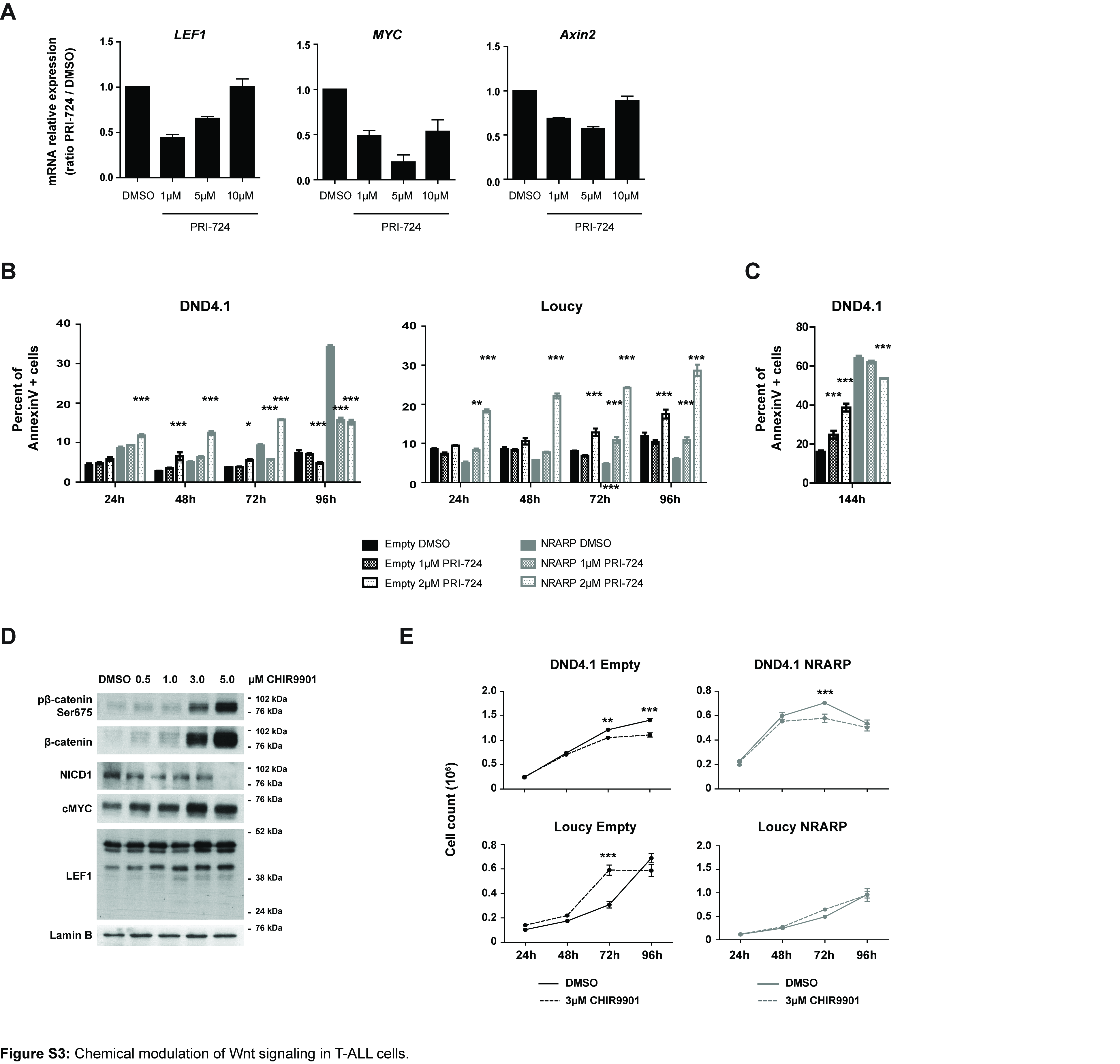

Supplement: Supplementary file 5 — Supplementary Figure S3 [file 41388_2019_1042_MOESM5_ESM.tif]

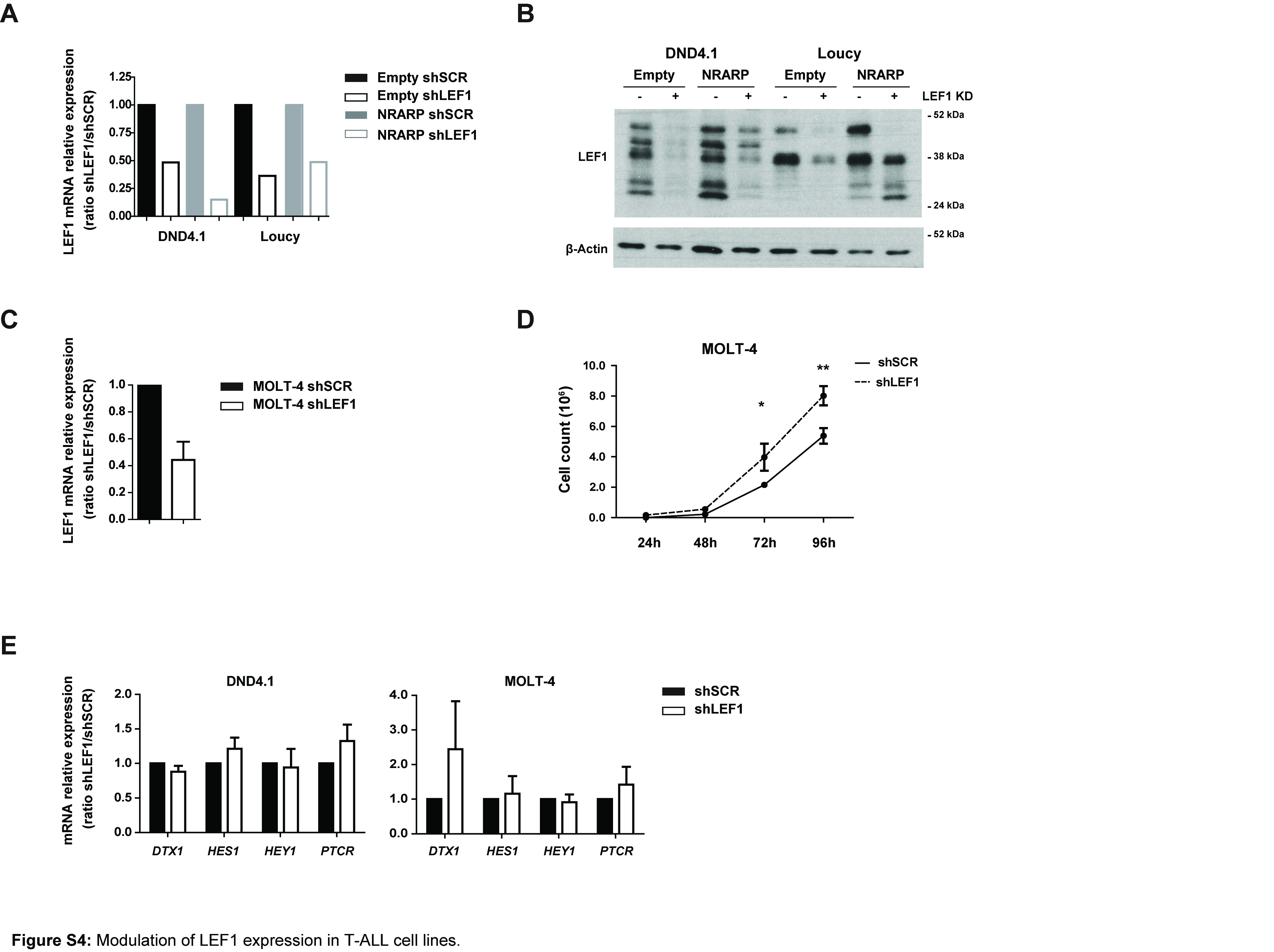

Supplement: Supplementary file 6 — Supplementary Figure S4 [file 41388_2019_1042_MOESM6_ESM.tif]

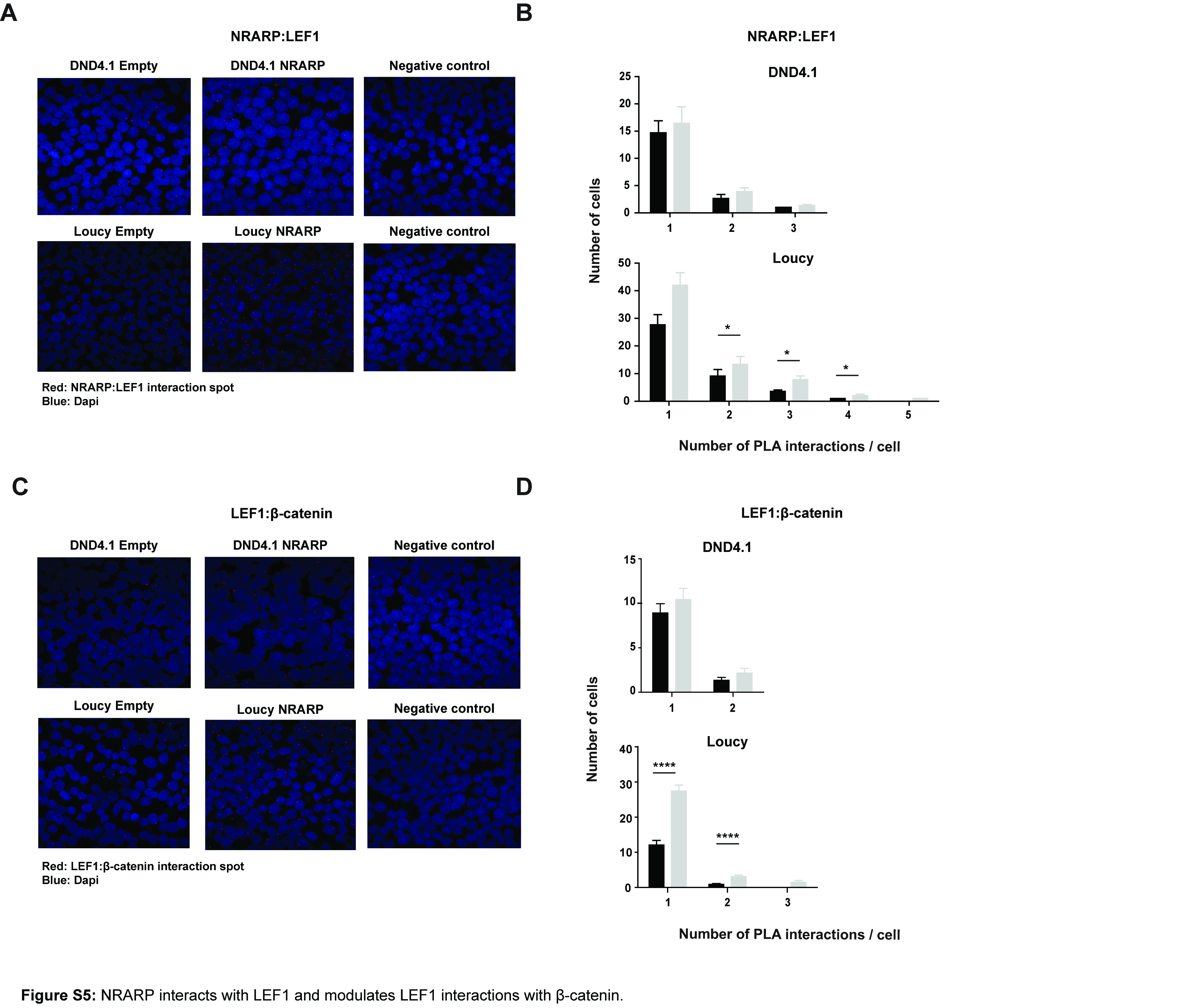

Supplement: Supplementary file 7 — Supplementary Figure S5 [file 41388_2019_1042_MOESM7_ESM.tif]

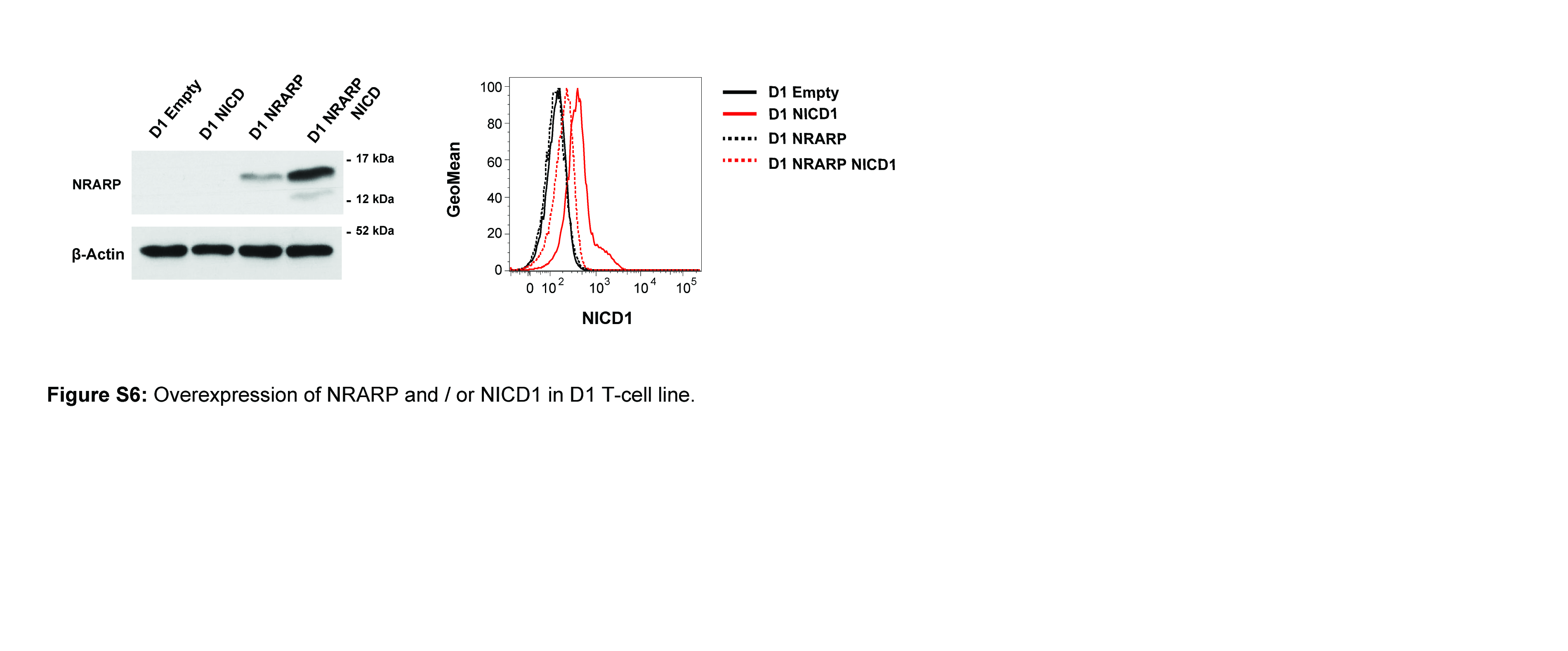

Supplement: Supplementary file 8 — Supplementary Figure S6 [file 41388_2019_1042_MOESM8_ESM.tif]

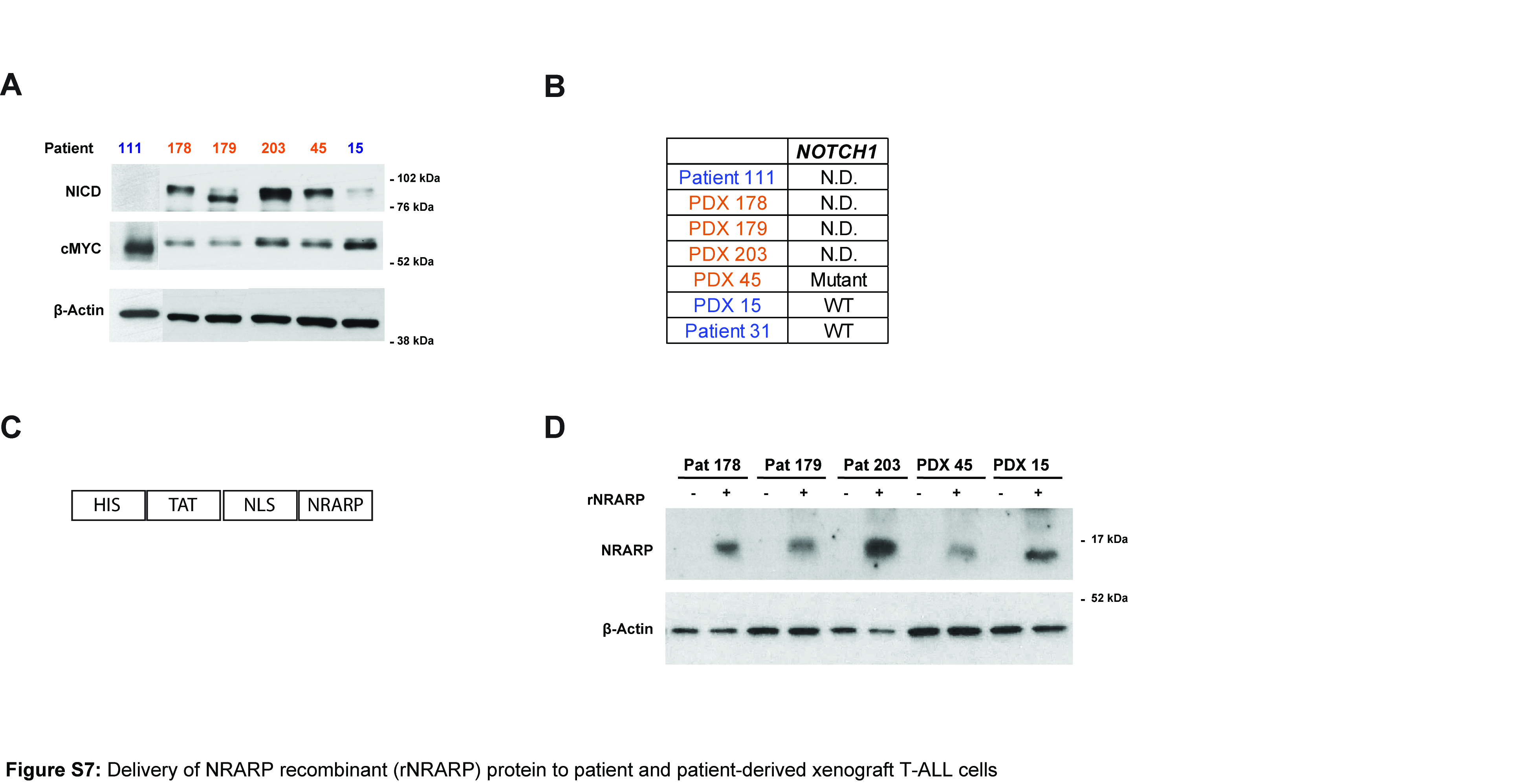

Supplement: Supplementary file 9 — Supplementary Figure S7 [file 41388_2019_1042_MOESM9_ESM.tif]
